# Comparative exome sequencing of metastatic lesions provides insights into the mutational progression of melanoma

**DOI:** 10.1186/1471-2164-13-505

**Published:** 2012-09-24

**Authors:** Jared J Gartner, Sean Davis, Xiaomu Wei, Jimmy C Lin, Niraj S Trivedi, Jamie K Teer, NISC Comparative Sequencing Program, Paul S Meltzer, Steven A Rosenberg, Yardena Samuels

**Affiliations:** 1 The Cancer Genetics Branch, National Human Genome Research Institute, National Institutes of Health, Bethesda, MD, 20892, USA; 2 The Genetics Branch, National Cancer Institute, National Institutes of Health, Bethesda, MD, 20892, USA; 3 Division of Laboratory and Genomic Medicine, Department of Pathology and Immunology, Washington University School of Medicine, St Louis, MO, USA; 4 Genetic Disease Research Branch, National Human Genome Research Institute, National Institutes of Health, Bethesda, MD, 20892, USA; 5 NIH Intramural Sequencing Center, National Human Genome Research Institute, National Institutes of Health, Bethesda, MD, 20892, USA; 6 The Surgery Branch, National Cancer Institute, National Institutes of Health, Bethesda, MD, 20892, USA; 7 Genome Technology Branch, National Human Genome Research Institute, National Institutes of Health, Bethesda, MD, 20892, USA

## Abstract

**Background:**

Metastasis is characterized by spreading of neoplastic cells to an organ other than where they originated and is the predominant cause of death among cancer patients. This holds true for melanoma, whose incidence is increasing more rapidly than any other cancer and once disseminated has few therapeutic options. Here we performed whole exome sequencing of two sets of matched normal and metastatic tumor DNAs.

**Results:**

Using stringent criteria, we evaluated the similarities and differences between the lesions. We find that in both cases, 96% of the single nucleotide variants are shared between the two metastases indicating that clonal populations gave rise to the distant metastases. Analysis of copy number variation patterns of both metastatic sets revealed a trend similar to that seen with our single nucleotide variants. Analysis of pathway enrichment on tumor sets shows commonly mutated pathways enriched between individual sets of metastases and all metastases combined.

**Conclusions:**

These data provide a proof-of-concept suggesting that individual metastases may have sufficient similarity for successful targeting of driver mutations.

## Background

Cancer is mainly a genetic disease with mutations arising that can either activate proto-oncogenes or inactivate tumor suppressor genes. The incidence of malignant melanoma is increasing worldwide. In fact, the most recent statistics predict approximately 69,000 new diagnoses and 8,700 deaths in the coming year in the United States alone [1]. Once melanoma has metastasized it has an extremely poor prognosis, with 5 year relative survival of just 15% [2]. In the past decade many genetic alterations have been discovered that influence tumor growth and spread. The knowledge gained during this time has led to the recent approval of the BRAF inhibitor PLX4032 (Zelboraf^TM^) by the FDA for treatment of late–stage melanoma [3].

The recent advances in next-generation sequencing have allowed for the discovery of new causal variants and have also afforded us the opportunity to ask new questions which could help dictate future treatment strategies. In this study we use whole exome sequencing to investigate two sets of distinct metastases to determine similarity.

## Results/discussion

### Exome sequencing and analysis

We performed whole exome sequencing of two distinct metastases from two individuals with melanoma (Additional file 1: Table S1). In both patients, metastatic deposits were present in multiple anatomic sites, as is typical for this form of cancer. Genomic DNA samples derived from these metastases underwent whole exome re-sequencing in parallel with their matched normal DNA. Exonic sequences were enriched with Agilent's SureSelect technology for targeted exon capture [4], targeting 50 Mb of sequence from exons and flanking regions in nearly 20,000 genes. Sequencing was performed with the Illumina GAII platform, and reads were aligned using ELAND (Illumina, Inc., San Diego, CA) followed by cross_match (http://www.phrap.org) to the reference human genome (Build 36.1). On average, 11.7 Gb of sequence were generated per sample to a mean depth of 103X to achieve exome builds with at least 87.5% of the targeted bases covered by high quality genotype calls. To eliminate common germ line mutations from consideration, we filtered variants observed in dbSNP130 or in a high quality set of common variants from the 1000 genomes project. To determine which of these alterations were somatic, we compared variants identified in the metastasis to their matched normal tissue and removed any variants found in the normal. From these putative alterations, 2356 potential somatic mutations in 1256 different genes were identified in the samples sequenced.

**Figure 1:**
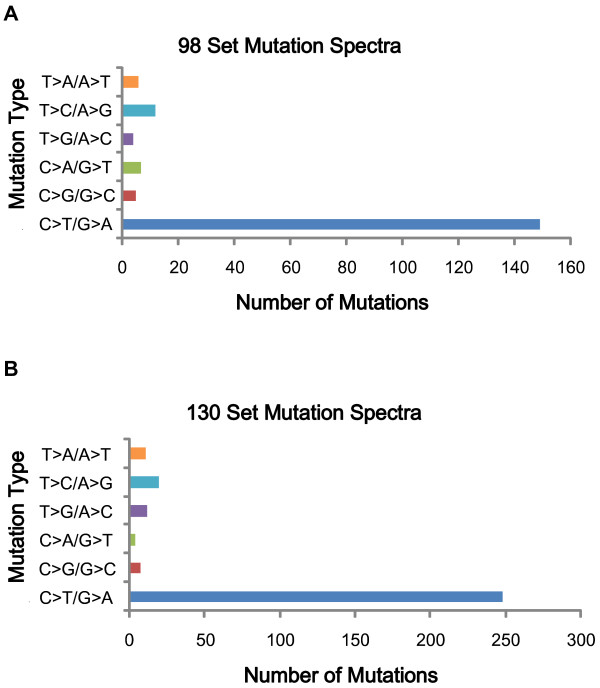
**Whole exome capture and sequencing analysis of melanoma samples derived from two sets of paired metastatic tumors.** Schematic overview of mutation identification approach of the four whole exomes. Samples were obtained at The Surgery Branch, National Cancer Institute.

A major challenge of such studies is discriminating true mutations from the large number of possible sequence alterations identified. Previously determined criteria were applied to discriminate between these possibilities [5]. Briefly, a MPG score of 10 or greater and a MPG/coverage ratio above 0.5 at a given position in both matched tumors as well as the normal was required to reliably evaluate an alteration. Once this criterion was applied ~68% of the potential alterations were removed, leaving 750 somatic base substitutions in 686 genes for further scrutiny. A total of 663 mutations were heterozygous alterations and 87 were loss of heterozygosity (Additional file 2: Table S2 & Additional file 3: Table S3). Of these alterations, 490 caused amino acid changes (non-synonymous), including 453 that were missense, 28 nonsense and 5 occurring at splice sites. There were 260 silent (synonymous) substitutions. A total of 3 small deletions and 1 insertion were observed. For a schematic of our analysis see Figure 1.

For each of the somatic mutations identified in the lesions derived from the two patients, we determined whether the same somatic mutation was present in anatomically distinct metastases from the same patient. The majority of the identified mutations were present in both samples from a given patient (mean of 97%, range 96-98%); (Tables 1 &2). These data propose that most of the somatically acquired mutations in these cancers occur before the development of metastatic lesions and might be considered potential founder mutations. Other identified mutations were present in one of the metastases examined, but not the second metastasis (mean of 2.75%, range 1.46- 4.04%) (Tables 1 &2). Many genes previously implicated in melanoma or other cancer types were seen to be mutated including the melanoma BRAF [Swiss-Prot: P15056] mutation V600E which was found in both metastases in the 98 set (Additional file 2: Table S2). Also observed in this set were shared mutations in MAPK10 [Swiss-Prot: P53779] which has been suggested to be a candidate tumor suppressor [6], BRCA1 [Swiss-Prot: P38398] [7], as well as ERBB4 [Swiss-Prot: Q15303] [8], GRIN2A [Swiss-Prot: Q12879] [5], and GRM3 [Swiss-Prot: Q14832] [9] which have previously been reported to be mutated in melanoma. In the 130 set of metastases we find a frame shift mutation shared by both in PTEN [Swiss-Prot: P60484] (Additional file 2: Table S2), a known tumor suppressor [10]. Mutations in genes proposed to play a role in cancer were also shared in these metastases including ADAM29 [Swiss-Prot: Q9UKF5] [11], ADAM19 [Swiss-Prot: Q9H013] [12], and NOTCH2 [Swiss-Prot: Q04721] [13].

**Table 1:** Summary of differences between metastases in 98 set

**Sample Set**	**Non-synonymous (NS)**	**Synonymous (S)**	**NS + S**	**Found in 14T only (NS)**	**Found in 14T only (S)**	**Found in 98T only (NS)**	**Found in 98T only (S)**	**Found in both Mets (NS)**	**Found in both Mets (S)**	**% found in both Mets (NS)**	**% found in both Mets (S)**	**% found in both Mets (NS + S)**
98 set	185	87	272	4	1	3	3	178	83	96.22	95.40	95.96

Table shows total number of mutations found for each set. NS- non-synonymous mutation; S- synonymous mutation; NS + S total mutations both non-synonymous and synonymous.

**Table 2:** Summary of differences between metastases in 130 set

**Sample Set**	**Non-synonymous (NS)**	**Synonymous (S)**	**NS + S**	**Found in 130T only (NS)**	**Found in 130T only (S)**	**Found in 133T only (NS)**	**Found in 133T only (S)**	**Found in both Mets (NS)**	**Found in both Mets (S)**	**% found in both Mets (NS)**	**% found in both Mets (S)**	**% found in both Mets (NS + S)**
130 set	305	173	478	3	3	0	1	302	169	99.02	97.69	98.54

Table shows total number of mutations found for each set. NS- non-synonymous mutation; S- synonymous mutation; NS + S total mutations both non-synonymous and synonymous.

**Figure 2:**
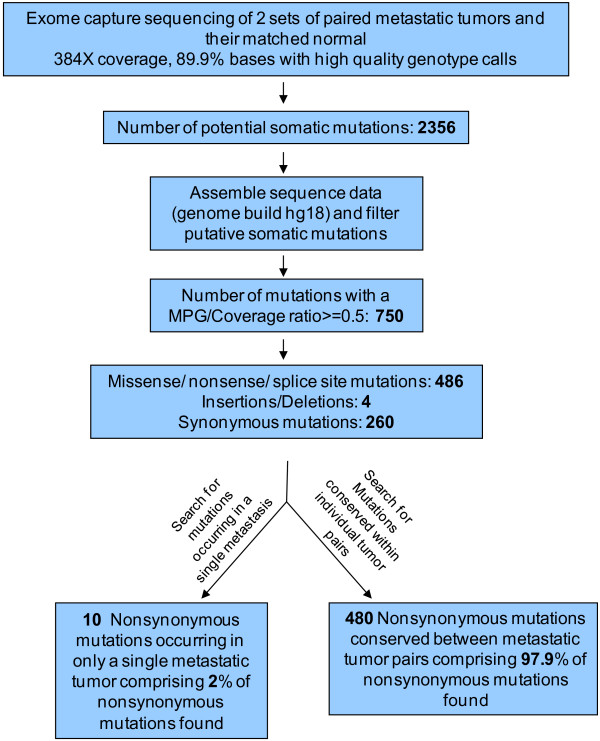
**Mutation spectra of single base pair substitutions in melanoma whole exome sequencing.** The number of each of the six classes of base substitutions resulting in non-synonymous changes in the whole exome screen is shown. **A.**) Mutation spectra from 98 set; **B.**) Mutation spectra from 130 set.

Our observed somatic mutations could either be ‘driver’ mutations that have a role in melanoma neoplasia or ‘passenger’ changes that are functionally inert. In this whole exome screen, we identified 490 non-synonymous and 260 synonymous mutations, yielding a ratio of non-synonymous to synonymous changes (N/S ratio) of ~1.9:1; which is not higher than the N/S ratio of 2:1 predicted for non-selected passenger mutations [14], suggesting that most of these alterations are likely to be ‘passenger’ mutations. The number of C > T mutations was significantly greater than the numbers of other nucleotide substitutions, resulting in a high prevalence of C:G > T:A transitions (*P* < 0.001) (Figure 2). Finally, a total of 13 dinucleotide substitutions were observed, of these, 12 were CC > TT/GG > AA changes, all consistent with the previously documented ultraviolet light exposure signature [15].

### Copy number analysis

**Figure 3:**
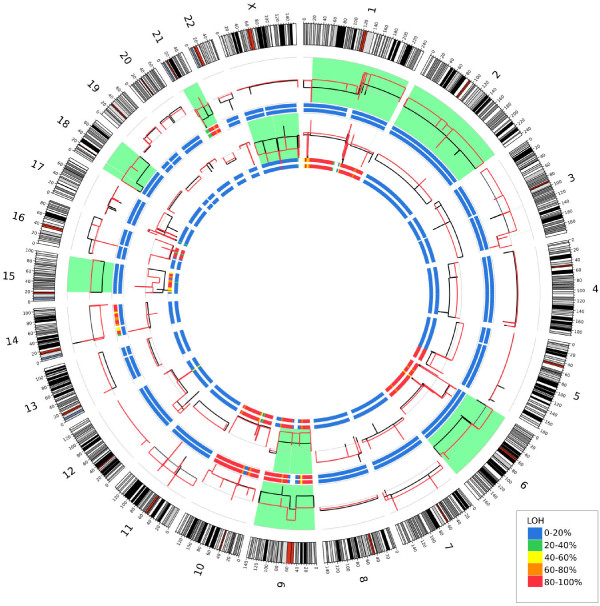
**Circos plot depicting copy number in the genome.** Outer ideogram runs clockwise from chromosome 1 to chromosome X with labels in Mb of physical distance. The data are represented in several tracks. The innermost track is a heat map representing the proportion of variants showing LOH in 5 MB bins for the 130 metastatic sample. The next innermost track shows the LOH results for the 133 metastatic sample. The data tracks depict relative copy number for the 130 met sample (in red) and the 133 met sample (in black). Regions that show large copy number differences between the two metastatic samples are highlighted in light green. Proceeding outward are two more heatmap LOH tracks for sample 14 met and then for sample 98 met. Finally, relative copy number profiles for samples 14 (in black) and 98 (in red) are shown; again, light green highlights regions of significant copy number change. For all heatmap tracks, blue represents no LOH in the region while red represents nearly all SNVs in the region showing LOH.

We then used a novel approach to determine copy number variants (CNV) from our exome data using aligned reads for each tumor and normal pair. Genomic windows which were defined by reading blocks of a fixed number of reads in the normal sample allowed the generation of a pseudo-CGH data output as described in the Methods section. Validation of this methodology was performed to determine accuracy. A selection of genes identified as having copy number differences in the 130 set was analyzed using qPCR and this analysis yielded the results expected in 14 of 16 cases or 87.5% (Additional file 4: Figure S1). Using this methodology we were able to compare our metastatic pairs to their matched normal, as well as one metastasis to the other. Through this analysis we were able to show similar, concordance between 2 matched metastatic sets with a 10% and 35% difference between the 130 set and 98 set metastases, respectively (Table 3 and Figure 3). Our CNV data identified copy number changes in previously reported regions of amplification and deletion in melanoma, such as amplification of 6p, a region previously shown to contain VEGF amplifications [16]. Also seen were copy number losses in both arms of chromosome 10 (both metastatic pairs) as well as loss in both arms of chromosome 9 (130 set and 14T sample) which have been previously reported in 40 to 50% of melanoma samples [17]. Chromosome 9p contains the CDKN2A [Swiss-Prot: P42771] locus, which is a region known to encode tumor suppressor proteins p16^INK4^ and p19^ARF^[18]. Loss of heterozygosity was also determined for each sample by identifying SNVs that were heterozygous in the normal sample for loss in the tumor. To do this, we characterized the SNV in the tumor as heterozygous (no LOH) or homozygous (LOH) and calculated a percentage of LOH for each sample in each region Figure 3. Interestingly, differences can be seen with apparent LOH not accompanied by any CNV differences. These regions of copy neutral LOH (CN-LOH) have been seen in solid cancers before [19], where it has been proposed that errors in mitosis, double strand break repair, or exposure to chemotherapeutic agents may provide a mechanism for induction [20].

**Table 3:** Percent copy number difference

**Set name**	**% Copy number difference between the two metastases**
130	10.86
98	34.95

Difference between the two metastases for each set: Total Mb different were calculated as described in methods at an absolute value of 0.35. The % difference was calculated by taking the total Mb difference and dividing by the total genome size (build hg18).

### Pathway enrichment analysis

**Figure 4:**
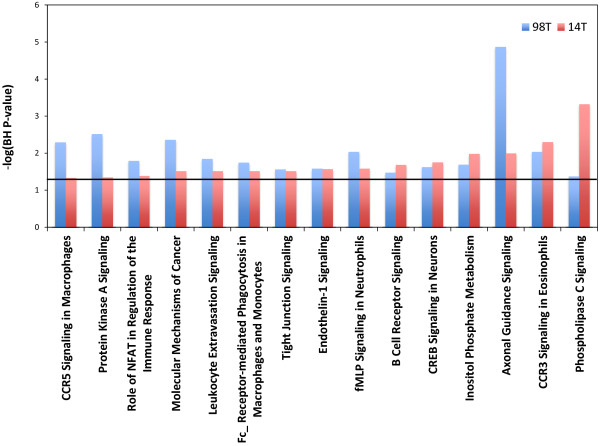
**Shared enriched pathways in the 98 set.** Y axis represents the –log p-value for enrichment. Black horizontal line represents a –log value of 1.3.

**Figure 5:**
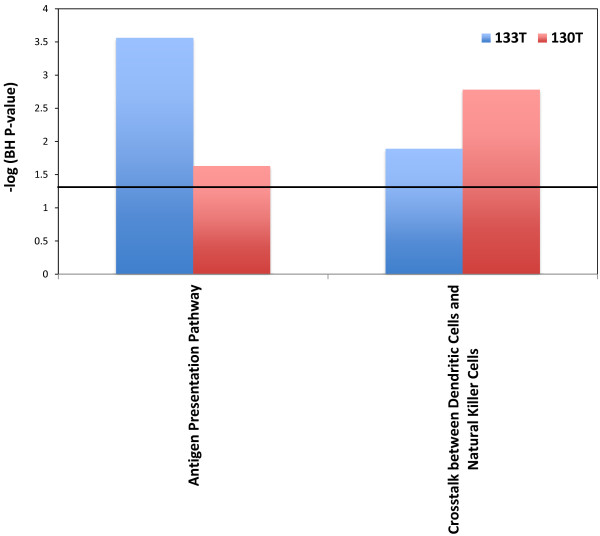
**Shared enriched pathways in the 130 set.** Y axis represents the –log p-value for enrichment. Black horizontal line represents a –log value of 1.3.

**Figure 6:**
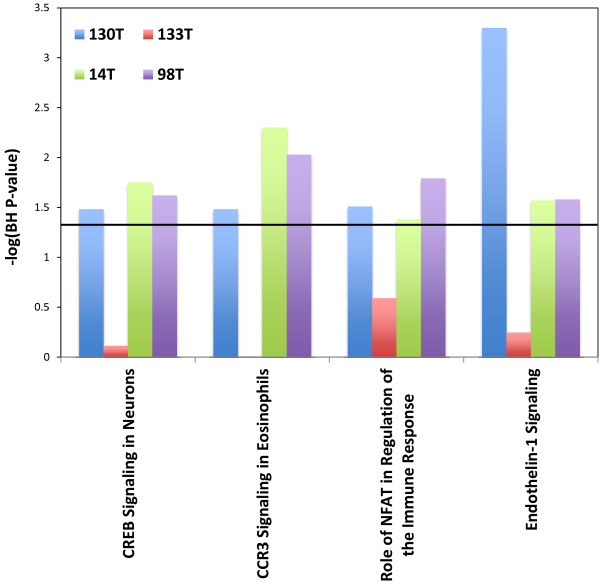
**Shared enriched pathways between the two sets.** Y axis represents the –log p-value for enrichment. Black horizontal line represents a –log value of 1.3.

Merging our SNV data with our CNV data we then performed pathway analysis to search for enriched pathways in our metastases. This analysis showed several pathways to be significantly enriched and shared between metastases in our 98 set (Figure 4). For our 130 set there were only 2 enriched pathways shared between the two metastases (Figure 5). This is due to fact that only two pathways were enriched in 130T using this analysis. When compared across tumors there were no pathways found to be enriched in all tumors from both sets, once again due to the lack of enriched pathways in sample 130T. There were however 4 pathways that were found to be enriched in 3 of the samples (Figure 6). Among these, Endothelin-1 and CREB signaling have previously been suggested to play a role in melanoma [21,22]. Differences seen between enriched pathways in matched tumors could be attributed to copy number differences seen between the two samples due to large numbers of of genes being found in regions of difference. A complete list of pathways found to be enriched can be found in the Additional file 5: Table S4.

## Conclusion

Advances in next-generation Sequencing have allowed researchers to affordably generate vast amounts of data and address complex morbicentric genetics questions with incredible sensitivity and robustness. In the present study we used our data from whole exome sequencing of two distinct metastases from the same patient to elucidate the similarities and differences between the tumors. The answer to these questions could prove useful in selection of future treatment strategies.

Our data revealed similarities between the two paired metastases at the level of somatic mutation and copy number variations. These results would seem to indicate that in both sets the metastases were derived from the same parental clone that harbored the majority of the genetic alterations and chromosomal instability. The Clark model of melanoma progression holds that melanoma progression proceeds in a stepwise manner from melanocyte to melanoma, categorized by numerous molecular changes which facilitate the transition through each step [18]. Our results suggest that once the transition from the vertical growth phase to malignant melanoma occurs, a limited number of molecular changes subsequently arise.

Despite these findings, further investigation is warranted. Future work should include a side by side genetic analysis of primary tumors pertaining to the metastatic lesions as well as the analysis of more metastases pertaining to the same patients derived from different regions to see whether these results hold true. Nonetheless, these findings provide a proof-of-concept that sequencing of a limited number individual metastases may be sufficient for targeting of melanoma driver mutations.

## Methods

### Tumor tissues

Tissue and melanoma cell lines used for this study were described previously [23]. The clinical information associated with the melanoma tumors used in this study is provided in Additional file 1: Table S1.

### Exome capture

Exome capture was performed using the Sure Select Human All Exon 50 Mb System (Agilent Technologies, Santa Clara, CA). The manufacturer’s protocol for Sure Select Human All Exon System (Illumina Paired-End Sequencing Library Prep), version 1.0.1 was used, with the following modifications. Bioanalyzer steps were either performed using agarose gel or omitted. The Illumina library preparation portion of the SureSelect protocol was performed using the SPRIworks Fragment Library System (Beckman Coulter Genomics, Danvers, MA, USA) according to manufacturer’s protocols.

### Illumina sequencing

Sequencing was performed on the Illumina GAIIx platform with version 5 chemistry and version 5 flowcells according to the manufacturer’s instructions. 76 and 101 base paired-end reads were generated summary of sequencing statistics can be seen in Additional file 6: Table S5.

### Read mapping and variant analysis

Reads were initially aligned using ELAND (Illumina Inc., San Diego, CA). ELAND alignments were used to place reads in bins of approximately 100,000 base pairs. Unmapped reads were placed in the bin of the mate pair if the mate was mapped. Cross_match (Phil Green, http://www.phrap.org) was utilized to align the reads assigned to each bin to the corresponding ~100 kb of genomic sequence. Cross_match alignments were converted to the SamTools bam format, and then genotypes were called using bam2mpg ([24], http://research.nhgri.nih.gov). Bam2mpg was used to implement the Most Probable Genotype (MPG) algorithm, a Bayesian based method to determine the probability of each genotype given the data observed at that position. The quality score represents the difference of the log likelihoods of the most and second most probable genotype. The MPG was divided by the coverage at each position to calculate the MPG/coverage ratio.

To eliminate common germline mutations from consideration, alterations observed in dbSNP130 or in a high quality set of common variants from the 1000 genomes 11_2010 data release project were removed. To perform the 1000 genomes project filtering, low coverage genome data from 629 individuals was obtained from the November 2010 data release of the 1000 genomes project. From this list of variants we included those positions called by at least 3 of the 4 analysis methods used by the project. We further limited the list to those variants above 5% minor allele frequency. Polymorphisms were further removed by examination of the sequence of the gene in genomic DNA from matched normal tissue. Genotypes were annotated as described in [25].

Mutational analysis, confirmation, and determination of somatic status were carried out to validate all mutations found exclusively in one of the metastasis as previously described [8,23]. Sequence traces of the Validation Screen were analyzed using the Mutation Surveyor software package and all genes had 93% coverage or above (SoftGenetics, State College, PA).

### Copy number variation analysis

Copy number estimates for the tumor were generated using aligned reads for each tumor and normal pair (http://github.com/seandavi/ngCGH). Genomic windows were defined by reading blocks of a fixed number of reads (default 1000 reads) in the normal sample. Within each defined genomic window, the number of reads in the tumor was quantified. For each genomic window, a log2 ratio was calculated between the number of reads in the tumor and the number of reads in the normal. The window size mean, median, and median absolute deviation (MAD) as well as the total number of windows are summarized in Additional file 7: Table S6. Note that these windows are not defined in regards to capture regions but are distributed across the genome. The resulting pseudo-CGH data output was segmented using the circular binary segmentation (CBS) algorithm as implemented in the DNAcopy Bioconductor package (Venkatraman E. Seshan and Adam Olshen, DNAcopy: DNA copy number data analysis. R package version 1.25.1.). The segmented data were used as input to the Circos genome plotting algorithm [26]. Segmented regions showing an absolute difference greater than 0.35 (in log2 units) between the two metastasis sample segmentation results were considered to represent different copy number as a log2 ratio of 0.35 of above represents 3 standard deviations when estimated by the derivative log ratio spread (DLRS) defined as:

$$\frac{IQR\sum_{i=2}^{n}r_{i}-r_{i-1}}{1.34\times \sqrt{2}}$$

Where r_i_ is the log2 ratio for the i^th^ window and n is the total number of windows. The denominator is just a normalization parameter to make DLRS the same scale as the standard deviation (when calculated on a normally-distributed random variable).

### Copy number validation

The results of CNV pseudo-CHG array were used to create a Nexus Copy Number file (BioDiscovery) and genes were randomly selected showing a CNV difference between either one or both of the samples and the normal. Primers for the gene indicated were designed using primer 3 with Line-1 primers used as controls for normalization (Additional file 8: Table S7). 1 ng of each sample gDNA was mixed with 2× Fast SYBR Green PCR mix at a final volume of 10 μl in triplicate (Applied Biosystems cat # 4355612). qPCR analysis was done using the ABI 7900HT Fast Real-Time PCR system (with a standard program of stage 1: 50°C for 2 min; stage 2: 95°C for 10 min; stage 3: 40 cycles of 95°C for 15 s and 60°C for 1 min). Results were analyzed using Microsoft Excel and SPSS.

### Pathway analysis

CNV and SNV data were combined and IPA (Ingenuity ®Systems, http://www.ingenuity.com) was used to investigate pathway enrichment for each tumor and set. The resulting p-values were adjusted for multiple testing via the benjamini-hochberg procedure. Adjusted p-values less than 0.05 were considered significant. Common pathways between tumors were merged using SequelPro and MySQL. Pathways in common between two tumor types that were significant were plotted using the ggplot2 package in *R*.

## Competing interests

The authors declared that they have no competing interests.

## Authors’ contributions

JJG and YS designed the study; JEG, and SAR collected and analyzed the melanoma samples; NISC performed the exome capture, sequencing, and exome analysis; JJG, SD, XW, JCL, NST and JKT analyzed the genetic data. JJG SD and YS wrote the paper. All authors read and approved the final manuscript.

## Supplementary Material

Additional file 1 **Table S1.** Is a table listing the relevant clinical information for the patients in each set. Click here for file

Additional file 2 **Table S2.** Is a complete list of variants found in our whole exome capture of the 98 set. Click here for file

Additional file 3 **Table S3.** Is a complete list of variants found in our whole exome capture of the 130 set. Click here for file

Additional file 4 **Figure S1.** Contains Results of qPCR validation. Click here for file

Additional file 5 **Table S4.** Is a complete list of pathways found to be enriched in each individual tumor sample. Click here for file

Additional file 6 **Table S5.** Is a summary of sequencing statistics for each tumor. Click here for file

Additional file 7 **Table S6.** Is summary of pseudo-CGH windows used for deriving copy number from our exome sequencing. Click here for file

Additional file 8 **Table S7.** Is complete list of primers used to evaluate our CNV data trough real time PCR. Click here for file
