# Supplementary material for: Comparative exome sequencing of metastatic lesions provides insights into the mutational progression of melanoma
Source: BMC Genomics. 2012 Sep 24;13:505. doi: 10.1186/1471-2164-13-505 (PMC3500261; doi:10.1186/1471-2164-13-505)
Supplement: Additional file 4 — Figure S1. Contains Results of qPCR validation. [file 1471-2164-13-505-S4.pdf]

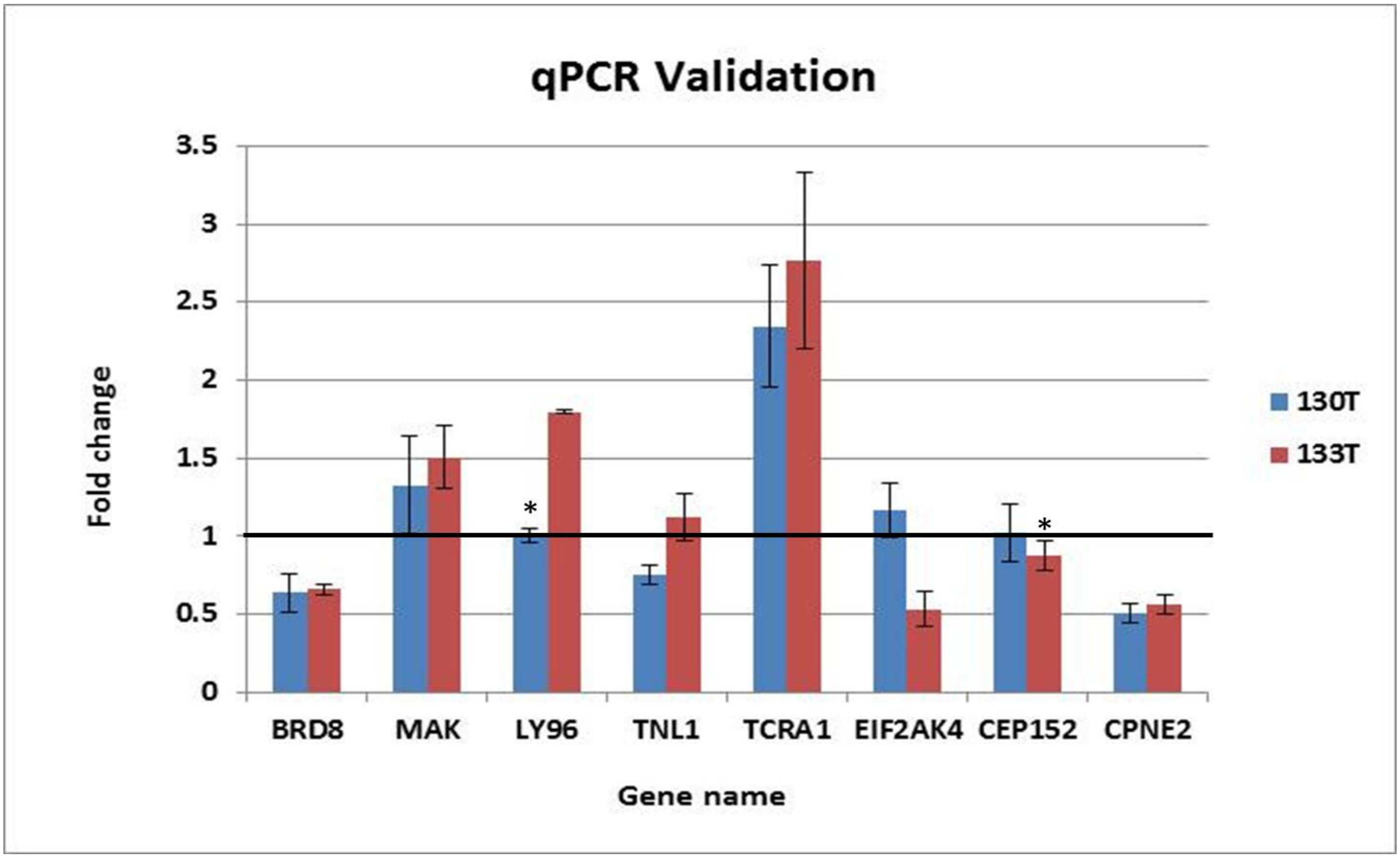

Supplementary Figure 1: Results of qPCR validation: 8 genes and their copy number status determined using qPCR to validate pseudo-CGH analysis. Our pseudo-CGH analysis showed in the gene: BRD8 a loss in both samples; MAK a gain is expected in both samples, LY96 a gain in both samples; TNL1 a loss in 130T; TCRA1 a gain in both samples; EIF2AK4 a loss in 133T; CEP152 loss in 133T; CPNE2 a loss in both samples. An * indicates sample where qPCR results do not validate the pseudo-CGH results. Fold change is indicated on the Y axis with 1 representing the normal samples copy number.
